# The effect of a natural polyphenol supplement on iron absorption in adults with hereditary hemochromatosis

**DOI:** 10.1007/s00394-022-02829-8

**Published:** 2022-03-23

**Authors:** Simone Buerkli, Laura Salvioni, Natalie Koller, Christophe Zeder, Maria José Teles, Graça Porto, Jana Helena Habermann, Irina Léa Dubach, Florence Vallelian, Beat M. Frey, Diego Moretti, Jeannine Baumgartner, Michael B. Zimmermann

**Affiliations:** 1 Laboratory of Human Nutrition, Institute of Food Nutrition and Health, Department of Health Science and Technology, Swiss Federal Institute of Technology (ETH Zurich), LFV D27.2, Schmelzbergstrasse 7, CH8092 Zurich, Switzerland; 2 Clinical Pathology, S. João University Hospital Center, Porto, Portugal; 3 Clinical Hematology, Santo António Hospital, Porto University Hospital Center (CHUP), Porto, Portugal; 4 Abel Salazar Institute for Biomedical Sciences (ICBAS), Porto, Portugal; 5 Institute of Research and Innovation in Health Sciences (i3S) of the University of Porto, Porto, Portugal; 6 Division of Internal Medicine, University Hospital of Zurich, Zurich, Switzerland; 7 Blood Transfusion Service, Swiss Red Cross, Schlieren, Switzerland; 8 Present Address: Department of Health, Swiss Distance University of Applied Sciences, Regensdorf/Zurich, Switzerland

**Keywords:** Polyphenols, Iron absorption, Hereditary hemochromatosis, Reducing dietary iron absorption, Supplement

## Abstract

**Objectives:**

We developed a natural polyphenol supplement that strongly chelates iron in vitro and assessed its effect on non-heme iron absorption in patients with hereditary hemochromatosis (HH).

**Methods:**

We performed in vitro iron digestion experiments to determine iron precipitation by 12 polyphenol-rich dietary sources, and formulated a polyphenol supplement (PPS) containing black tea powder, cocoa powder and grape juice extract. In a multi-center, single-blind, placebo-controlled cross-over study, we assessed the effect of the PPS on iron absorption from an extrinsically labelled test meal and test drink in patients (*n* = 14) with HH homozygous for the p.C282Y variant in the *HFE* gene. We measured fractional iron absorption (FIA) as stable iron isotope incorporation into erythrocytes.

**Results:**

Black tea powder, cocoa powder and grape juice extract most effectively precipitated iron in vitro. A PPS mixture of these three extracts precipitated ~ 80% of iron when 2 g was added to a 500 g iron solution containing 20 µg Fe/g. In the iron absorption study, the PPS reduced FIA by ~ 40%: FIA from the meal consumed with the PPS was lower (3.01% (1.60, 5.64)) than with placebo (5.21% (3.92, 6.92)) (*p* = 0.026)), and FIA from the test drink with the PPS was lower (10.3% (7.29 14.6)) than with placebo (16.9% (12.8 22.2)) (*p* = 0.002).

**Conclusion:**

Our results indicate that when taken with meals, this natural PPS can decrease dietary iron absorption, and might thereby reduce body iron accumulation and the frequency of phlebotomy in patients with HH.

Trial registry: clinicaltrials.gov (registration date: 9.6.2019, NCT03990181).

**Supplementary Information:**

The online version contains supplementary material available at 10.1007/s00394-022-02829-8.

## Introduction

Hereditary hemochromatosis (HH) is characterized by increased iron accumulation in tissue and organs, potentially leading to liver cirrhosis, hepatocellular carcinoma, diabetes, arthropathy, and heart disease [1]. It is one of the most common genetic diseases in Caucasian populations, mainly of Nordic or Celtic ancestry [2–6]. *HFE*-related HH accounts for 85–90% of all cases associated to the homozygous p.C282Y mutation [2]. Iron overload in *HFE*-related HH is caused by relative hepcidin deficiency [7]. This is due to the conformation of HFE, which affects the signaling pathway that regulates hepcidin expression [8]. The reduction in hepcidin expression leads to inappropriately high dietary iron absorption [7–10].

The standard of care for HH patients is phlebotomy to reduce accumulated body iron [11–13]. The morbidity and mortality of HH patients is significantly reduced when the treatment is started before the development of cirrhosis and/or diabetes [14, 15]. The frequency of the phlebotomies depends on iron status, determined by measuring transferrin saturation (TSat) and serum ferritin (SF)[5]. Frequent phlebotomies can be inconvenient and a burden for many patients [16].

Previous studies reported equivocal effects of dietary iron intake (heme and non-heme) on iron status in HH [17–20]. A systematic review of studies assessing the effect of diet in HH concluded that despite limited evidence, dietary modification may be a beneficial adjunct strategy to limit iron accumulation [21]. Dietary modification would require limiting intake of heme and non-heme iron, as well as reducing the intake of iron absorption enhancers (e.g., vitamin C) and increasing intake of iron absorption inhibitors, such as phytate and polyphenols (PP). PP are widely distributed among plants and inhibit non-heme iron absorption by forming insoluble Fe–PP complexes in the intestinal lumen [22]. Polyphenol-rich dietary sources have been shown to be inhibitory in various iron absorption studies [22–28]. In patients with HH, the consumption of black tea with a meal led to a significant reduction in iron absorption from a single meal. Furthermore, HH patients in the tea drinking group, who consumed black tea with all main meals over 1 year, had a smaller increase in SF than the control group who consumed water with meals [29]. Therefore, patients with HH would most likely benefit from the regular intake of a PP-rich supplement to reduce body iron accumulation and, therefore, frequency in required phlebotomies. However, the inhibitory effect of PP on iron absorption is strongly dependent on the PP structure [22]. A supplement containing silybin, a flavonoid extracted from milk thistle (*Silybum marianum*), reduced serum iron response in HH patients in an oral iron tolerance test [30], whereas a supplement containing proanthocyanidins did not [31].

The objectives of this study were: (1) to develop a natural PP supplement with high efficiency in chelating iron in vitro, and (2) to determine its inhibitory effect on iron absorption when provided with a non-heme iron-rich test drink and test meal in HH patients. Using in vitro digestion and measuring iron solubility, we screened various PP-rich dietary sources to formulate a natural PP supplement (PPS) with maximum iron-chelating potential. The PPS was formulated in capsules and administered to patients with HH. Fractional iron absorption (FIA) was measured using stable iron isotopes. The comparison of FIA from the drink to the meal further allowed assessing the effect of the meal matrix. We hypothesized that iron absorption from an iron-fortified test drink and from a non-heme iron-rich test meal given with the PPS will be reduced significantly compared to when consumed with the placebo capsules.

## Methods

### Food analyses

Polyphenol-rich dietary sources were selected based on a high content of gallic acid equivalents (GAE) according to data extracted from Phenol-Explorer [32–34], a low content of ascorbic acid (based on literature), and their availability in dried powder form. These were: black tea powder, cinnamon, chestnut flour, cloves, cocoa powder, coffee common sage, grape juice extract, marjoram, oregano, star anise, and turmeric. All foods were purchased from food or drug stores, if required, they were milled in-house to obtain a homogenous powder. Total PP concentrations of all dietary sources and the PPS mixture were measured with a modified Folin–Ciocalteau method [35]. Concentrations were measured as GAE. Total iron (Fe) concentrations were measured by graphite furnace atomic absorption spectrophotometry (GFAAS, AA240Z; Varian), after complete mineralization of the sample by microwave digestion (MLS TurboWave; MLS GmbH). Total phytic acid (PA) concentrations were measured by a modified method by Makower [36]. Vitamin C content of the test meal was analyzed via HPLC (Waters Acquity H-Class) after stabilization and extraction in metaphosphoric acid and reduction via ditiothreitol [37].

### Analysis of iron precipitation by polyphenol-rich dietary sources in vitro

The in vitro digestion method was used to determine the effect of the PP-rich dietary sources on solubility and bioaccessibility of iron. We mixed different doses of the dietary source rich in PP (60, 120, or 180 mg) into a 30 g solution (nanopure water) containing 600 µg Fe (20 µg Fe/g) as FeSO_4_. The tested doses of the PP-rich dietary sources were equivalent to 1 g, 2 g and 3 g powder in relation to a 500 g solution containing 10 mg Fe (matching the test condition in stable iron isotope study). In vitro digestion was performed in triplicate samples and each condition was tested twice. Using amylase (Takadiastase from *Aspergillus oryzae*, Sigma-Aldrich) samples (30 g sample with 200 mg amylase) underwent oral digestion for 10 min, followed by a pH reduction with 6 M HCl to pH 2. A pepsin (from porcine gastric mucosa, Sigma-Aldrich) solution (800 mg in 10 mL 0.1 M HCl) was added to simulate gastric digestion in a 37 °C shaking water bath. After 2 h, the pH was increased with 0.5 M KOH and a pancreatin (bile extract, from porcine and pancreatin from porcine pancreas, both from Sigma-Aldrich) solution (80 mg pancreatin, 500 mg bile extract in 20 ml 0.1 M NaHCO_3_) was added. After 10 min of intestinal digestion, all samples were centrifuged at 2000 g for 15 min. The iron concentration of the supernatant was measured using GFAAS (Fe_soluble_), whereas the total iron concentration (Fe_total_) was calculated based on measured concentrations of the dietary source. We assumed that PP–Fe are insoluble complexes and precipitate during the centrifugation; therefore, we calculated Fe_insoluble_ = Fe_total−_Fe_soluble_. The PPS was formulated based on the three dietary sources that exhibited the highest capacity to chelate iron in vitro (online resource Table S1). By mixing these three PP rich sources, the accumulated daily intake of each PPS ingredient is lower, and a blend of three components might be more effective in recusing iron absorption.

### Stable iron isotope study participants and study sites

Study participants (*n* = 14) were patients with diagnosed HH, all homozygous in the p.C282Y variant in the *HFE* gene. Other inclusion criteria were: Between 18 and 65 years of age, weight of below 80 kg, body mass index (BMI) within 18.5–25 kg/m^2^, the last phlebotomy being at least 4 weeks prior to first test meal/drink administration, and expected to comply with the study procedures. Exclusion criteria were: pregnancy and breastfeeding, acute illness or infection, metabolic or chronic diseases, use of long-term medication and consumption of mineral and vitamin supplements 2 weeks prior and during the study period, a scheduled phlebotomy during the study period, and participation in any other clinical study within the last 30 days.

The study was conducted at the Santo Antonio Hospital—Porto University Hospital Center (CHUP), in Porto, Portugal and at the Laboratory of Human Nutrition at ETH in Zurich, Switzerland. The study arm in Portugal was conducted between August 2019 and October 2019 and the study arm in Switzerland between December 2019 and August 2020. The study was performed according to the Declaration of Helsinki, and ethical approval for the study was provided by the ethical review committees of ETH Zürich and the Canton of Zürich (BASEC 2019-01776) in Switzerland, and of the Porto University Hospital Center (CHUP), Portugal (2019.127(107-DEFI/111-CE)). The study was registered at clinicaltrials.gov (NCT03990181). Informed signed consent was obtained from all the participants.

### Study design and procedures

**Fig. 1:**
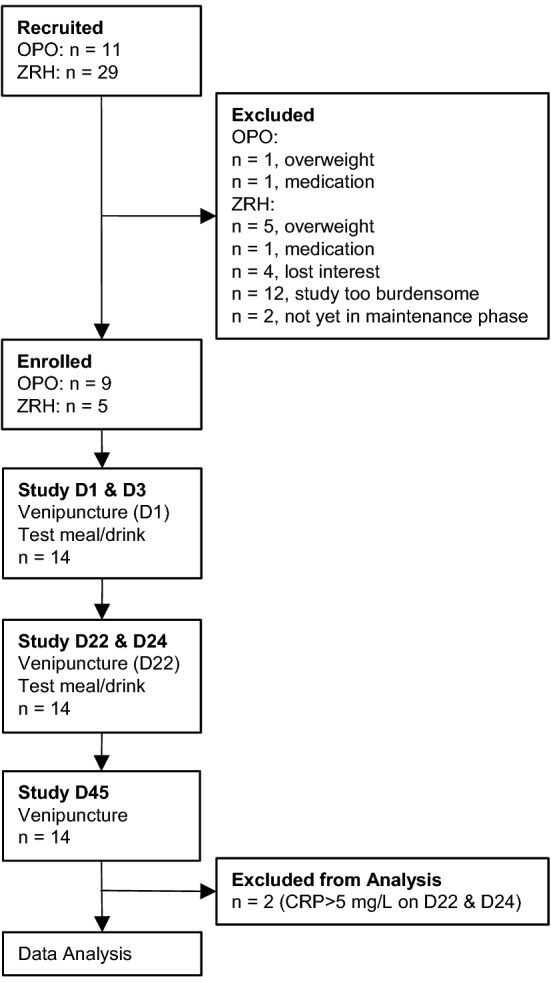
Study participant flow

This was a multi-center, partially randomized, single-blind, placebo-controlled cross-over study (Fig. 1). The study included four experimental conditions: (1) extrinsically labelled test meal (containing 8 mg native Fe and 2 mg ^57^Fe as ferrous sulfate [FeSO_4_]) consumed with 2 g of the PPS (Meal-PPS); (2) extrinsically labelled test meal (containing 8 mg native Fe and 2 mg ^58^Fe as FeSO_4_) consumed with 2 g of maltodextrin (placebo) (Meal-Placebo); (3) extrinsically labelled test drink (water fortified with 8 mg Fe of natural isotopic composition as FeSO_4_ and 2 mg ^57^Fe as FeSO_4_) consumed with 2 g of the PPS (Drink-PPS); and (4) extrinsically labelled test drink (water fortified with 8 mg Fe of natural isotopic composition as FeSO_4_ and 2 mg ^57^Fe as FeSO_4_) consumed with the placebo supplement (Drink-Placebo). We decided to provide a PPS dose of 2 g to a 500 g meal/drink based on the results from the in vitro digestion experiments (online resource Table S1).

Study participants were recruited at their regular phlebotomy visit at the Santo Antonio Hospital—Porto University Hospital Center (CHUP) in Porto, Portugal, the Department of Internal Medicine of the University Hospital Zurich, and the Blood Transfusion Service Zurich in Switzerland. In Porto, if a patient was interested, informed consent was obtained and a questionnaire administered to assess in- and exclusion criteria approximately 1–2 months before study day 1. In Zurich, if a patient was interested, informed consent was obtained and in- and exclusion criteria were checked in a screening on study day 1. On the study days, all participants were fasting, meaning no food intake after 8 pm and no drinks after midnight on the evening before. In the morning of study days 1, 3, 22 and 24, participants came to the study center for administration of the experimental conditions Meal-PPS, Meal-Placebo, Drink-PPS, or Drink-Placebo. The order of the experimental condition was partially randomized, meaning the same stable iron isotope was not consumed within the same study week.

Venous blood samples were collected by venipuncture on study days 1, 22, and 43 for analysis of hemoglobin (Hb) concentration, and indices of iron status and inflammation. On study days 1 and 22, we further measured serum hepcidin, and on study days 22 and 43 we determined stable iron isotope incorporation into erythrocytes.

### Composition of test meals and drinks

The test meals (Meal-PPS and Meal-Placebo) consisted of four slices of whole grain toast bread (Olivers Toast, Vollkorn, Migros) (115 g), 48 g cashew paste (Cashewmus, Alnatura), two bean tarts (80 g), and 44 g apricot-pumpkinseed fruit leather. The toast, cashew paste, and all ingredients for the bean tarts and fruit leather were purchased in bulk and frozen until use. The bean tarts and the fruit leather were prepared in one batch and frozen until use. Each bean tart contained a prebaked wheat flour pastry case (Törtchenbödeli, Midor, Migros), blended canned white beans (Soisson Bohnen, MClasic, Migros), egg yolks (53 g + , free-range, Migros), ground peeled almonds (Mandeln gemahlen geschält, MClassic Migros), and refined sugar (Feinkristallzucker, Migros) (at a ratio of 15:32:32:24:13). The fruit leather contained apricots and pumpkinseeds in a ratio of 10:3, which were homogenized with water, and thin layers of the purée were dried for 6 h at 80 °C. The test meals were provided to the participants together with deionized water. Each test meal portion contained 8.02 ± 0.005 mg native Fe, 1.06 ± 0.06 g phytic acid, 26.9 ± 0.1 mg vitamin C, and 300.8 ± 17.4 mg total polyphenols (gallic acid equivalents). The test drinks (Drink-PPS and Drink-Placebo) consisted of 470 g deionized water mixed with 30 g FeSO_4_ solution containing 8 mg Fe of natural isotopic composition and 2 mg labelled Fe. Total weight of the test meal (including drinking water and water for rinsing the isotopes) and of the test drink was 500 g. Thus, the ratio of PPS dose to meal/drink amount corresponded to the ratio in the in vitro experiments.

### Preparation and administration of stable iron isotopes, test meals, drinks and PPS

We labelled FeSO_4_ with ^57^Fe-enriched and ^58^Fe-enriched elemental iron (with 95.56% and 99.89% isotopic enrichment, respectively, all Chemgas, Boulogne-Billancourt, France) as previously described [38]. The FeSO_4_ solutions were pre-weighted in individual doses into Teflon vials and stored at 4 °C until use. The PPS mixture was prepared in one batch by mixing milled black tea powder, cocoa powder and grape juice extract in equal parts. Black, 000 sized gelatin capsules were manually filled with 1 g (± 5 mg) of the PPS mixture, or maltodextrin for the placebo supplements (Maltodextrin 6, Nutricia GmbH). The meals were thawed the night before administration, and the toast slices were heated before consumption and spread with the cashew paste. The isotopically labelled FeSO_4_ solution (providing 2 mg iron) was poured directly onto the bread of the test meals, or provided diluted in water in a small glass with the test drinks. The vial was rinsed twice with 1 ml water, which was also poured onto the bread, or into the glass. Participants were asked to start by taking two capsules of the PPS or placebo supplement and then to consume the test meal or drink. The isotope solution was consumed at once halfway through the drink. The glass containing the isotope solution was rinsed twice with 10 g of water and consumed by the participant.

### Blood analyses

Venous blood samples were collected and immediately processed after withdrawal. EDTA whole blood was used for the analysis of Hb on the sampling day using an automated hematology analyzer (Sysmex XE-5000 analyzer, Sysmex Corporation, both study sites). Heparinized whole blood was aliquoted for analysis of isotopic composition. Blood collected in serum tubes was centrifuged, and serum samples aliquoted for SF analysis (Porto: Elecsys Ferritin assay; Roche Cobas analyzer; Zurich: Immulite; Siemens Healthcare Diagnostics), and for determination of C-reactive protein (CRP), alpha-1-acid glycoprotein (AGP) and soluble transferrin receptor (sTfR) using a multiplex ELISA method [39]. Serum iron (SFe) and total iron binding capacity (TIBC) were measured using colorimetry, and sHep using a commercial ELISA Kit (DRG Hepcidin 25, DRG Instruments GmbH). All aliquoted samples collected in Portugal were stored at − 20 °C until shipment on dry ice to ETH Zurich and further stored at − 20 °C until analysis. TSat was calculated using the formula SFe/TIBC*100. Acute inflammation was defined as CRP concentrations > 5 mg/L or alpha-1-acid glycoprotein (AGP) > 1 g/L.

We determined FIA and calculated the amounts of ^57^Fe and ^58^Fe isotopic labels in blood on study days 22 and 43 based on the shift in iron isotope ratios in erythrocytes and on the estimated amount of iron circulating in the body [40]. We performed the analyses by multicollector-inductively coupled plasma mass spectrometry (MC–ICP–MS, Neptune; Thermo Finnigan) as previously described [41]. We calculated circulating iron in the body based on Hb and blood volume, derived from the participant’s height and weight [42], and assuming an 80% incorporation of absorbed iron into erythrocytes [40].

### Sample size calculation and statistical analysis

A priori we calculated a sample size of 18 to be adequate to detect a 50% reduction in fractional iron absorption from the iron-rich meal/drink when consumed with the PPS, taking into account a standard deviation of 0.4, and a probability of an α error of 0.05 to reach a power β of 0.8. These assumptions were based on data from iron absorption studies performed in HH and healthy individuals [24, 29]. A 50% reduction in iron absorption was estimated to be a relevant reduction. To account for dropouts, we anticipated a sample size of 20. After 14 participants had completed the study, we ran an interim analysis. The data of these participants reached a power of 0.799 and 0.862 to explain our hypothesized differences in FIA from the iron-enriched meals and drinks, respectively, when consumed with the PPS compared to placebo.

We used IBM SPSS statistics (Version 24) for statistical analysis. To test for differences in the percentage of precipitated Fe after in vitro digestion between the PP doses (1, 2 and 3 g), a one-way ANOVA with post hoc Bonferroni correction was run for each PP source, and the PPS. A repeated-measures ANOVA was performed with percentage of precipitated Fe as the dependent variable, the dose as the repeated measure and the PP source as between-subject factor. Post hoc Bonferroni corrected comparisons were made to test for significant differences between the PP sources. We assessed the correlation of precipitated iron with total PP concentration using Spearman’s rho test. We tested data of the human absorption study for normality by Shapiro–Wilk tests. Normally distributed data are presented as means ± standard deviation (SD), log transformed normal data as geometric mean with 95% confidence interval (95%CI), and non-normal data as median and interquartile range (IQR). We tested between-group differences in FIA using log-transformed data with dependent samples *T* tests. Predictors of iron absorption were estimated using linear regression with log FIA as dependent variable, and sex (1: female, 2: male), meal matrix (1: meal, 2: drink), intake of PPS (1: PPS, 2: placebo), log SF, log sHep, and log CRP as independent variables, reported are standardized β. The α-level of significance was set at 0.05.

## Results

### In vitro* precipitation of iron by different dietary sources of PP and doses*

**Fig. 2:**
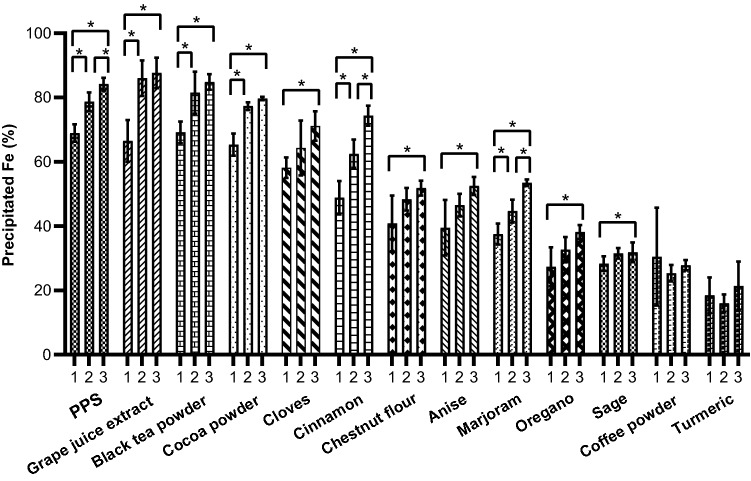
Percentage of precipitated iron after in vitro digestion in a 20 µg Fe/g solution with the PPS and other food powders at different doses of 1 g, 2 g and 3 g. Shown are means ± SD. Significant differences between doses within a PP source are indicated with * (one-way ANOVA with Bonferroni corrections, *p* < 0.05)

**Fig. 3:**
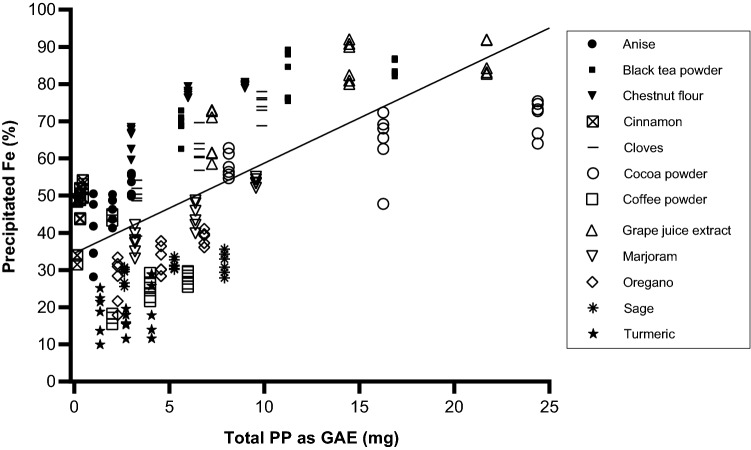
Correlation of percentage precipitated iron with measured PP content (mg) as gallic acid equivalent (GAE). Spearman’s rho = 0.629, *p* < 0.001 (1-tailed)

We found differences in percentage of precipitated iron in vitro between different PP sources (*p* < 0.001) and between PP doses (*p* < 0.001), as well as a significant PP source x dose interaction (*p* < 0.001) indicating that the dose–effect was dependent on the PP source. Results of the post hoc comparisons are shown in the online resource Table S1. Grape juice extract, black tea powder and cocoa powder showed the strongest ability to complex iron, with no significant differences between them. When 2 g of their powders was digested in a 500 g solution containing 20 µg/g Fe, 77–86% of the total iron was precipitated (online resource Table S1, and Fig. 2). All other PP-rich dietary sources had a significantly lower ability to complex iron (all at 2 g): Cloves and cinnamon precipitated around 62–64%, chestnut flour, anise and marjoram around 44–48%, oregano, common sage, and coffee powder around 25–32%, and turmeric around 15% (online resource Table S1, and Fig. 2). For black tea powder, grape juice extract, and cocoa there was a significant increase in precipitated Fe from 1 to 2 g of the PP source, but not when the doses were increased from 2 to 3 g (online resource Table S1). The PPS containing grape juice extract, black tea powder and cocoa powder led to a 78.7 ± 2.9% reduction in soluble iron when 2 g of the PPS underwent in vitro digestion in 500 g solution containing 20 µg/g Fe (Fig. 2, and online resource Table S1). The PPS mixture showed a significant increase in precipitated Fe from dose 1–2 g, and from 2 to 3 g (online resource Table S1, and Fig. 2). There was a moderately strong positive relationship of total PP concentrations (as GAE) of the dietary PP source (online resource Table S2) with their ability to precipitate iron (*r*_s_ = 0.629, *p* < 0.001) (Fig. 3).

### Characteristics of the PP-rich supplement

The PP concentration of the PPS was 129 ± 3.41 mg GAE/g, and in cocoa powder, grape juice extract, and black tea powder the measured concentrations were 41.4 ± 1.20 mg GAE/g, 178.7 ± 8.97 mg GAE/g, and 135.9 ± 3.82 mg GAE/g, respectively. The measured iron concentration of the PPS was 183.6 ± 15.3 µg/g Fe, and PA concentration was 1.13 ± 0.091 mg/g. Therefore, 2 g of the PPS contained 259.15 ± 6.82 mg total PP (as GAE), 367.22 ± 30.64 µg Fe, and 2.26 ± 0.18 mg PA.

### Characteristics of the test meal

#### Human study participants

Among the approached HH patients in Porto, 11 interested participants were recruited, and nine of these fulfilled all inclusion criteria. Among the HH patients in Zurich, 29 participants were recruited, and five of these fulfilled all inclusion criteria. We were able to enroll and complete the study with 14 participants. On study day 22, an increased CRP (CRP > 5 mg/L) was measured in two participants; therefore, their FIA data of days 22 and 24 were not included in the analysis (Fig. 1). Of these 14 study subjects, seven were female and seven male. Participant characteristics, baseline anthropometric measurements, indices of inflammation, iron status and hepcidin concentrations are shown in Table 1.

**Table 1:** Study participant characteristics, baseline anthropometric measurements and iron status based on samples from study days 1 and 22

	All	Female	Male
*n*	14	7	7
Age (years)^a^	44.8 ± 9.86	43.0 ± 9.9	46.6 ± 10.2
Weight (kg)	68.1 ± 10.6	60.7 ± 8.1	75.5 ± 7.0
Height (cm)	172.6 ± 12.7	161.7 ± 4.9	183.6 ± 6.7
BMI (kg/m^2^)	22.8 ± 2.4	23.2 ± 2.8	22.4 ± 2.2
CRP (mg/L)^b^	0.75 (0.32, 1.74)	0.88 (0.17, 4.51)	0.63 (0.21, 1.93)
AGP (g/L)	0.57 ± 0.19	0.57 ± 0.15	0.56 ± 0.25
Hb (g/dL)	15.4 ± 1.57	14.2 ± 0.80	16.6 ± 1.18
SF (ng/mL)^c^	69.8 (54.4–114.6)	59.0 (43.5–74.6)	86.0 (65.0–223.5)
sTfR (mg/L)	3.99 (3.61–4.37)	3.96 (3.70–4.19)	4.00 (3.33–5.37)
SFe (µg/dL)	164.5 ± 36.7	164.4 ± 32.5	164.5 ± 43.2
TIBC (µg/dL)	308.0 (282.7–335.6)	307.9 (281.7–400.2)	308.0 (283.0–314.6)
TSat (%)	50.41 (44.11, 57.61)	49.2 (40.0, 60.6)	51.6 (40.9, 65.2)
sHep (ng/mL)	3.49 (2.26, 5.37)	2.95 (1.53, 5.69)	4.13 (1.97, 8.67)

BMI, body mass index; Hb, hemoglobin; SF, serum ferritin; CRP, C-reactive protein; AGP, α1-acid glycoprotein; sHep, serum hepcidin; sTfR, soluble transferrin receptor; SFe, serum iron; TIBC, total iron binding capacity; TSat, transferrin saturation

^a^Values are mean ± standard deviation, all such values

^b^Values are geometric means and 95% confidence interval, all such values

^c^Values are median and interquartile range, all such values

#### Iron absorption

**Fig. 4:**
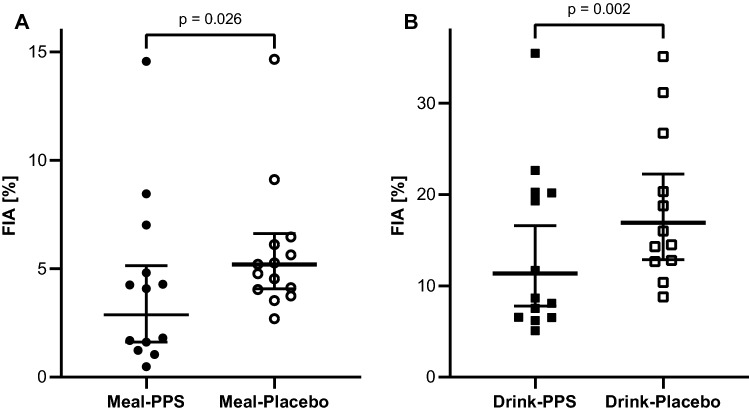
**A** FIA from test meals and **B** test drinks consumed either with the PPS (Meal-PPS & Drink-PPS) or with the placebo supplement (Meal-Placebo & Drink-Placebo). Shown are individual datapoints and the geometric mean with the 95% CI, *p* = 0.026, and 0.002, respectively, paired samples *T* test

The geometric mean and 95% CI of the total iron absorption from the meal with and without the PPS (Meal-PPS vs. Meal-Placebo) was 0.313 (0.167, 0.586) mg Fe and 0.522 (0.393, 0.693) mg Fe, respectively (*p* = 0.035). FIA from the meal with and without the PPS (Meal-PPS vs. Meal-Placebo) was 3.01% (1.60, 5.64) and 5.21% (3.92, 6.92), respectively (*p* = 0.026) (Fig. 4A). Without the meal matrix, the total iron absorption from the drink with and without the PPS (Drink-PPS vs. Drink-Placebo) was 1.07 (0.756, 1.52) mg Fe and 1.69 (1.28, 2.22) mg Fe, respectively (*p* = 0.003). FIA from the drink with and without the PPS (Drink-PPS vs. Drink-Placebo) was 10.3% (7.29 14.6) and 16.9% (12.8 22.2), respectively (*p* = 0.002) (Fig. 4B). The intake of the PPS led to a 42 and 39% reduction in FIA in both, meals (*p* = 0.026) and drinks (*p* = 0.002), respectively. We identified a meal matrix effect (Drink-Placebo vs. Meal-Placebo) which was independent from the effect of the polyphenol supplement, and led to a 69% reduction in FIA (*p* < 0.001). The pooled FIA geometric mean of both meals (3.96% (2.82, 5.56)) and both drinks (13.2% (10.5, 16.6)) also differed (*p* < 0.001) (70% reduction).

FIA in this study was independently predicted by meal matrix (*β* = 0.711, *p* < 0.001), hepcidin concentration (*β* = − 0.294, *p* = 0.008), sex (*β* = 0.278, *p* = 0.007, male had higher FIA), and PPS (*β* = 0.271, *p* = 0.002). These parameters explained 65% of the variability in FIA (*R*^2^ = 0.694, *R*^2^_adjusted_ = 0.653). In contrast, FIA was not associated with CRP (*β* = 0.081, *p* = 0.347) or ferritin (*β* = − 0.077, *p* = 0.530) (data not shown).

## Discussion

The main findings of this study are: (1) the natural polyphenol-rich dietary sources of the PPS complexed iron in vitro and reduced its solubility by ~ 80%; and (2) in the iron absorption study, the PPS reduced FIA from an iron-rich meal and drink by ~ 40% in adults with HH.

We tested the capability of various PP-rich dietary sources to precipitate iron *in vitro*. The selection of these sources was based on their measured total PP content as GAE. Coffee has a high concentration of total PP [43] and has shown to reduce iron absorption from a meal by 61% [22]. Nevertheless, in our in vitro experiments, coffee powder precipitated iron by 25–30%. This low precipitation was most likely due to the decreased solubility of the PPs in cold (room temperature and body temperature) water compared to coffee brewed in hot water. Cloves and cinnamon also showed strong capabilities to precipitate iron; however, these were not further investigated due to their high contents of eugenol [44] and coumarin [45]. Turmeric had the lowest capability to precipitate iron, which is in line with a human absorption study showing that iron absorption was not impaired by the addition of turmeric to a test meal [25]. Overall, the capability to precipitate iron in vitro correlated well with the total PP content of the dietary PP source. This is in agreement with the results from an iron absorption study showing that the reduction in FIA correlates with total PP content [24].

The effect size of the PPS in our study (40%) is smaller compared to a previously reported 70% reduction in FIA from a meal consumed with black tea in HH patients [29] and compared to the 60–90% reduction in iron absorption from a meal consumed with either black tea, coffee, cocoa, or peppermint tea in healthy subjects [24]. This difference might be attributed to the following mechanisms: First, the administration method of the PPS in gelatin capsules might have led to a delay in their release and, therefore, an incomplete mixture of the PPS with the test meal (compared to administration of PP-rich beverages). To solve this issue, other galenic forms, such as a pressed tablet, might overcome the delay in release due to encapsulation. Furthermore, taking the supplement 15 min before each meal (rather than at the start of the meal) might improve food/PPS mixing in the gastrointestinal tract. Second, in prepared tea and beverages, the polyphenols are already dissolved from their matrix and are available to form PP–Fe complexes. The polyphenols from our PPS required to be first dissolved in the stomach before being able to form PP–Fe complexes. Third, we administered a 2 g mixture of powdered Ceylon tea, cocoa and a grape juice extract, which contains a broad range of different polyphenolic structures.

The iron absorption measured in our study in homozygous p.C282Y patients with HH was overall lower than reported by Kaltwasser et al. [29] who measured iron absorption in a similar patient group from a meal which was consumed with either tea or water. The meal consisted of beef, rice, spinach and potatoes, and was consumed with 1.5 g Ceylon tea, extracted for 5 min in 250 mL water. The FIA from this meal with tea was 6.9% compared to 22.1% when consumed with water, which would result in a ~ 70% reduction in FIA [29]. These measured FIA values are higher compared to the FIA values from our test meal consumed with the PPS (3.0%) and placebo (5.2%). The overall lower FIA from the test meal in our study might be attributed to the high content in PA and PP. The meal matrix per se had a highly inhibitory effect on FIA. It induced a significant reduction in FIA of ~ 60%, and was a main determinant of FIA in the logistic regression. The molar ratio of PA:Fe in the meal was 9.3:1, which is classified as highly inhibitory [46]. The major contributor of PA to the meal was the cashew paste, followed by toast bread > fruit leather > bean tarts. Besides the study by Kaltwasser et al., only few studies have measured iron absorption in HH patients using stable or radio iron isotopes, but all have reported higher iron absorption values. Iron absorption from ferric citrate in a chicken soup meal was reported to be 30% [47]; iron absorption from a composite meal containing wheat bread, hamburger, gravy, lettuce and tomato juice was reported to be 36.4% [48], and non-heme iron absorption from a meal containing beef, wheat bread, french fries and a vanilla milkshake was 41.3% [49]. An iron absorption study without a meal matrix reported 74% iron absorption from a ferric ascorbate reference dose [50]. In comparison, we measured 16.9% iron absorption without a meal matrix, and 10.3% iron absorption without a meal matrix but with the PPS. However, it has to be noted that these studies included patients diagnosed with HH before *HFE* was discovered, which may differ from our specified patient group being homozygous for the p.C282Y variant in *HFE*. Furthermore, our meal was highly inhibitory due to the high PA and native PP content, and during the preparation of the test drink containing FeSO_4_ iron hydroxide formation may have occurred.

According to Phenol-Explorer, flavanols make up for 72% of the total PP in black tea and 16% are accounted to hydroxybenzoic acids. In cocoa powder, flavanols are also the largest represented class and make up for 93% of the total PP in cocoa powder. The widest represented PP in grapes (*Vitis vinivera*) belong to the classes of anthocyanins (82%) and flavanols (16%) [32–34]. Therefore, the main represented components in our PPS can be accounted to flavanols. Flavanols contain catechol or galloyl groups, which are good proton donors and binding sites for ferric iron creating octahedral insoluble PP–Fe complexes [22, 24, 51].

The extent of iron accumulation in HH patients varies considerably (1.2–241 µg SF/L), and on average serum ferritin rises by 99 µg/L in a year [52]. Considering the following assumptions: our study test meal is consumed with the PPS three times per day; a mean Hb concentration of 15.4 g/dL; an average concentration of 3.47 mg Fe/g Hb; and 1 µg/L SF represents 8 mg of stored body iron [53, 54], the PPS could potentially lead to a yearly decrease of ~ 230 mg in absorbed iron, which would correspond to one unit of phlebotomized blood (450 mL). Or in other words, this is equivalent to ~ 29 µg SF and is 1/3rd of the average yearly increase in SF in HH patients [52]. However, the estimated mitigation in yearly SF increase is lower compared to the longitudinal study performed by Kaltwasser et al. [29], showing that regular tea drinking with all three main meals for 1 year led to a larger decrease in SF in the tea drinking group compared to the control group (77 µg SF difference) [29]. Nevertheless, the regular intake of our PPS with all main meals may be less cumbersome than brewing and regular tea drinking for some individuals.

A strength of our study is that, with the two experimental conditions of test drink and test meal, we have demonstrated that the PPS is able to reduce iron absorption by ~ 40% in both conditions, independently of the food matrix and other iron absorption inhibitors. The high PA content of our test meal may have weakened the effect of the PPS on FIA from a meal. A test meal with a lower PA content (but, therefore, also a lower non-hem iron concentration) would require measuring iron absorption from multiple meals, as stable iron isotopes need amounts in milligrams to be detectable and should not contribute more than 20% to the total iron content of the meal [55].

To summarize, our PPS has shown to reduce iron solubility in vitro by ~ 80%, and iron absorption in HH patients by ~ 40%, which could potentially correspond to the reduction of one phlebotomy session per year. However, to assess its efficacy, a placebo controlled longitudinal study would be required. There is increasing evidence suggesting that dietary PP may have a protective role against chronic diseases [56–58]. Thus, the additional daily intake of 6 g of the PPS (containing black tea powder, cocoa powder and grape juice extract) may provide additional health benefits for patients with HH. In conclusion, the intake of our PPS shows promise in reducing dietary iron absorption in patients with HH, and may also be of advantage in other iron overload diseases caused by excess dietary iron absorption.

## Supplementary Information

Below is the link to the electronic supplementary material.

Supplementary file1 (DOCX 28 kb)

## Data Availability

Data described in the manuscript, code book, and analytic code will be made available upon request pending application and approval.
