# Supplementary material for: The effect of a natural polyphenol supplement on iron absorption in adults with hereditary hemochromatosis
Source: Eur J Nutr. 2022 Mar 23;61(6):2967–77. doi: 10.1007/s00394-022-02829-8 (PMC9363374; doi:10.1007/s00394-022-02829-8)
Supplement: Supplementary file 1 — Supplementary file1 (DOCX 28 kb) [file 394_2022_2829_MOESM1_ESM.docx]

# Supplementary Information to:

# The effect of a natural polyphenol supplement on iron absorption in adults with hereditary hemochromatosis

Simone Buerkli^1^, Laura Salvioni^1^, Natalie Koller^1^, Christophe Zeder^1^, Maria José Teles^2^, Graça Porto^3,4,5^, Jana Helena Habermann^6^, Irina Léa Dubach^6^, Florence Vallelian^6^, Beat M. Frey^7^, Diego Moretti^1,8^, Jeannine Baumgartner^1^, Michael B. Zimmermann^1^

^1^ Laboratory of Human Nutrition, Institute of Food Nutrition and Health, Department of Health Science and Technology, Swiss Federal Institute of Technology (ETH Zurich), Zurich, Switzerland; ^2^ Clinical Pathology, S. João University Hospital Center, Porto, Portugal; ^3^ Clinical Hematology, Santo António Hospital - Porto University Hospital Center (CHUP), Porto, Portugal; ^4^ Abel Salazar Institute for Biomedical Sciences (ICBAS) Porto, Portugal; ^5^ Institute of Research and Innovation in Health Sciences (i3S) of the University of Porto, Portugal; ^6^ Division of Internal Medicine, University Hospital of Zurich, Zurich, Switzerland; ^7^ Blood Transfusion Service, Swiss Red Cross, Schlieren, Switzerland; ^8^ current affiliation: Swiss Distance University of Applied Sciences, Department of Health, Regensdorf/Zurich, Switzerland.

Corresponding author: Simone Buerkli; Laboratory of Human Nutrition, LFV D27.2, Schmelzbergstrasse 7, CH8092 Zürich; [simone.buerkli@hest.ethz.ch](mailto:simone.buerkli@hest.ethz.ch); +41 44 632 86 39

Table S1: Mean and standard deviation (SD) of the percentage of precipitated iron in a 20 µg Fe/g solution at different doses of the polyphenol-rich dietary sources (1, 2 and 3 g).

| **PP-rich dietary source** | **% precipitated Fe per dose** | | | **Differences between PP sources^c^** |
| --- | --- | --- | --- | --- |
|  | 1 g | 2 g | 3 g |  |
| PPS | 68.9 ± 2.7 A | 78.7 ± 2.9 B | 84.2 ± 2.0 C | - |
| Grape juice extract^a, b^ | 66.4 ± 6.6 A | 86.0 ± 5.5 B | 87.7 ± 4.7 B | a |
| Black tea powder | 69.0 ± 3.5 A | 81.7 ± 6.4 B | 84.8 ± 2.2 B | a |
| Cocoa powder | 65.3 ± 3.5 A | 77.5 ± 1.2 B | 79.7 ± 0.5 B | a |
| Cloves | 58.1 ± 3.3 A | 64.2 ± 8.7 AB | 71.1 ± 4.6 B | b |
| Cinnamon | 48.9 ± 5.2 A | 62.3 ± 4.3 B | 74.3 ± 3.2 C | b |
| Chestnut flour | 40.9 ± 8.6 A | 48.2 ± 3.5 AB | 52.0 ± 2.1 B | c |
| Anise | 39.5 ± 8.6 A | 46.5 ± 3.5 AB | 52.6 ± 2.8 B | c |
| Marjoram | 37.6 ± 2.3 A | 44.7 ± 3.5 B | 53.8 ± 1.1 C | c |
| Oregano | 27.3 ± 6.2 A | 32.8 ± 3.9 AB | 38.3 ± 2.1 B | d |
| Common sage | 28.0 ± 2.4 A | 31.6 ± 1.3 AB | 31.8 ± 3.0 B | d |
| Coffee powder | 30.6 ± 15.0 A | 25.3 ± 2.8 A | 27.9 ± 1.7 A | d |
| Turmeric | 18.6 ± 5.8 A | 15.9 ± 2.7 A | 21.1 ± 7.7 A | e |
| PP: Polyphenol; PPS: Polyphenol supplement.  ^a^ shown are means ± standard deviation (SD)  ^b^ different capital letters indicate significant differences between PP doses within each PP source (one-way ANOVA for each PP source, pairwise comparisons with Bonferroni correction), with p<0.05.  ^c^ different small form letters indicate significant differences between PP sources, (repeated measures ANOVA, pairwise comparisons with Bonferroni correction), with p<0.05. | | | | |

Table S2: Measured total PP concentration (mg GAE/g) of all PP-rich dietary sources (mean ± standard deviation).

| **PP-rich dietary source** | **Total PP (mg GAE/g)** |
| --- | --- |
| Grape juice extract | 120.6 ± 5.6 |
| Cloves | 135.5 ± 7.5 |
| Black tea powder | 93.7 ± 8.5 |
| Ceylon cinnamon | 54.8 ± 4.7 |
| Marjoram | 53.2 ± 2.9 |
| Cocoa powder | 49.9 ± 2.7 |
| Common sage | 43.9 ± 3.7 |
| Oregano | 38.0 ± 1.9 |
| Coffee | 33.2 ± 2.6 |
| Turmeric | 22.6 ± 4.1 |
| Star anise | 16.7 ± 0.9 |
| Chestnut flour | 2.5 ± 0.2 |
| GAE: Gallic acid equivalents; PP: Polyphenol. | |
